# Factors that regulate insulin producing cells and their output in *Drosophila*

**DOI:** 10.3389/fphys.2013.00252

**Published:** 2013-09-17

**Authors:** Dick R. Nässel, Olga I. Kubrak, Yiting Liu, Jiangnan Luo, Oleh V. Lushchak

**Affiliations:** Department of Zoology, Stockholm UniversityStockholm, Sweden

**Keywords:** insulin receptor, neuropeptide, metabolism, neuromodulation, insulin signaling, insulin release

## Abstract

Insulin-like peptides (ILPs) and growth factors (IGFs) not only regulate development, growth, reproduction, metabolism, stress resistance, and lifespan, but also certain behaviors and cognitive functions. ILPs, IGFs, their tyrosine kinase receptors and downstream signaling components have been largely conserved over animal evolution. Eight ILPs have been identified in *Drosophila* (DILP1-8) and they display cell and stage-specific expression patterns. Only one insulin receptor, dInR, is known in *Drosophila* and most other invertebrates. Nevertheless, the different DILPs are independently regulated transcriptionally and appear to have distinct functions, although some functional redundancy has been revealed. This review summarizes what is known about regulation of production and release of DILPs in *Drosophila* with focus on insulin signaling in the daily life of the fly. Under what conditions are DILP-producing cells (IPCs) activated and which factors have been identified in control of IPC activity in larvae and adult flies? The brain IPCs that produce DILP2, 3 and 5 are indirectly targeted by DILP6 and a leptin-like factor from the fat body, as well as directly by a few neurotransmitters and neuropeptides. Serotonin, octopamine, GABA, short neuropeptide F (sNPF), corazonin and tachykinin-related peptide have been identified in *Drosophila* as regulators of IPCs. The GABAergic cells that inhibit IPCs and DILP release are in turn targeted by a leptin-like peptide (unpaired 2) from the fat body, and the IPC-stimulating corazonin/sNPF neurons may be targeted by gut-derived peptides. We also discuss physiological conditions under which IPC activity may be regulated, including nutritional states, stress and diapause induction.

## Introduction

Insulin and IGF signaling (IIS) play pivotal roles during development and growth, but also in daily life of the mature organism where they regulate metabolism, reproduction, stress responses and other processes influencing aging and lifespan (Brogiolo et al., 2001; Giannakou and Partridge, 2007; Grönke et al., 2010; Teleman, 2010; Antonova et al., 2012; Itskov and Ribeiro, 2013). The molecular components of the IIS pathway are well-conserved over evolution, although the complexity is somewhat larger in mammals than in invertebrates, mainly due to increased numbers of genes encoding receptor types and downstream signaling elements (Brogiolo et al., 2001; Garofalo, 2002; Teleman, 2010). This review focuses on *Drosophila* and the reader is referred to some relevant reviews for other insects or invertebrates (Claeys et al., 2002; Kaletsky and Murphy, 2010; Antonova et al., 2012; Lapierre and Hansen, 2012; Badisco et al., 2013; Mizoguchi and Okamoto, 2013).

In *Drosophila* there are eight insulin-like peptides (ILPs), designated DILP1—8, but only one known receptor, dInR (Fernandez et al., 1995; Brogiolo et al., 2001; Grönke et al., 2010; Colombani et al., 2012; Garelli et al., 2012). The different DILPs are produced in various cell types and tissues in specific spatio-temporal patterns during development and in the adult fly and thus seem to play different functional roles. In the present account we will mainly deal with DILP function in the adult fly and only briefly discuss larval and developmental roles of these peptide hormones. Thus, we focus primarily on DILP2, 3, 5, 6, and 7 that have established roles in adult physiology, and highlight what is known about the regulation of production and release of these peptides. Of these, especially DILP2, 3 and 5 from the insulin producing cells (IPCs) of the brain have been the subject of many investigations. It should be made clear that whereas the functional roles of DILPs and IIS in *Drosophila* have been under intense study, the control of DILP production and release by extrinsic factors has only recently received some attention. There are on the other hand a large number of studies showing that genetic manipulations of IPCs affect transcription of the three brain-derived DILPs. We summarize here what is known about secreted factors, such as neurotransmitters, neuromodulators and hormones, that regulate activity in IPCs and thereby production and/or release of DILPs and co-expressed hormones. Also other factors and physiological conditions that affect IPC activity and DILP gene transcription will be discussed. As will be seen in this review there is a major gap in our knowledge on the integrated role of neuronal systems that regulate IPCs and physiological conditions requiring activation or inactivation of insulin signaling. In other words there is a need to identify conditions and signals that activate the different neuronal pathways that control IPCs.

## Anatomy and organization of insulin producing cells

**Figure 1:**
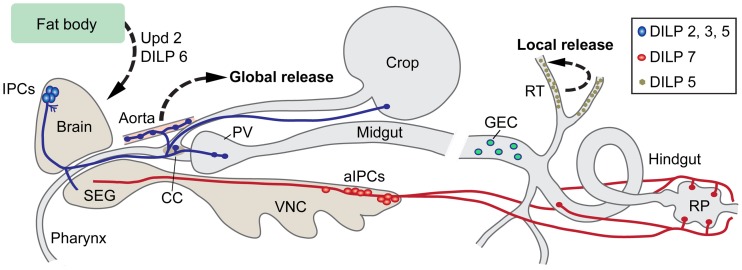
**Insulin-producing cells (IPCs) in the *Drosophila* nervous system and gut.** A set of 14 IPCs (blue) in pars intercerebralis of the brain send axons to the tritocerebrum (adjacent to the subesophageal ganglion, SEG), to the corpora cardiaca (CC) with associated aorta, proventriculus (PV), and the crop. Likely release sites for circulating *Drosophila* insulin-like peptides (DILPs) are in CC, aorta, PV, and crop. These IPCs produce DILP2, 3, and 5. The branches in protocerebrum (near cell bodies) and tritocerebrum of the brain could be dendritic and/or represent further release sites. A second set of 20 cells (aIPCs; red) is found in the abdominal neuromeres of the ventral nerve cord (VNC). These produce DILP7 and supply axon terminations to the hindgut including the rectal papillae (RP), and in females to reproductive organs (not shown here). At least two of the aIPCs send axons to the SEG. It is not clear whether their branches in the SEG are dendrites or release sites (or both). Additionally the principal cells (green) of the renal tubules (RT) produce DILP5. This DILP may act locally in the tubules. DILP6 is produced in fat body cells in the head and abdomen. The fat body also releases a leptin-like peptide, unpaired 2 (Upd2). Both DILP6 and Upd2 regulate IPC activity. In the midgut there are peptidergic endocrine cells (GEC) that may release peptides into the circulation to target cells in renal tubules and brain. This figure is updated and modified from Nässel (2012) which was partly based on Cognigni et al. (2011).

In larvae and adults of *Drosophila* DILP2, 3 and 5 are produced in a set of 14 median neurosecretory cells, IPCs, in the brain (Brogiolo et al., 2001; Cao and Brown, 2001; Rulifson et al., 2002; Geminard et al., 2009), *Dilp1* transcript was detected in larval IPCs (Rulifson et al., 2002), DILP7 in about 20 neurons of the abdominal neuromeres of the fused thoracic-abdominal ganglia (Brogiolo et al., 2001; Miguel-Aliaga et al., 2008; Yang et al., 2008) and DILP6 mainly in adipose cells of the fat body, but also in larval salivary glands and heart (Okamoto et al., 2009; Slaidina et al., 2009) (Table 1). These production sites are shown in Figure 1. Furthermore, DILP3 is produced in muscle cells of the adult midgut and DILP5 in follicle cells of the ovary as well as principal cells of the renal tubules (Brogiolo et al., 2001; Veenstra et al., 2008; Söderberg et al., 2011). DILP8 has been detected in the imaginal discs (bags of progenitor cells of adult tissues) of larvae (Colombani et al., 2012; Garelli et al., 2012). An early study employed *in situ* hybridization to reveal the following additional expression sites in the third instar larva: *Dilp2* in imaginal discs and salivary glands, and *Dilp4* in the embryonic anterior midgut and mesoderm (Brogiolo et al., 2001). Finally, DILP6, and maybe DILP2, are expressed in glial cells in the CNS of early larval stages where they play roles in neuroblast reactivation (Chell and Brand, 2010; Sousa-Nunes et al., 2011). Consulting the FlyAtlas gene expression database [flyatlas.gla.ac.uk; (Chintapalli et al., 2007)] there are no records for *Dilp1* and *4* expression in any larval or adult tissue, whereas the distribution of the other *Dilps* is largely confirmed. According to FlyAtlas *Dilp8* transcript is enriched in adult ovaries. The DILP1–8 expression patterns are summarized in Table 1.

**Table 1:** **Location of insulin-producing cells in *Drosophila***.

**DILPs**	**Location**		**Axon terminations**	**References**
	**Larvae**	**Adult**		
DILP 1	IPCs	–	–	Rulifson et al., 2002
DILP 2	IPCs	IPCs	Brain neuropil	Rulifson et al., 2002
	Imaginal discs		Corpora cardiaca	
	Salivary glands		Anterior aorta	
	Glial cells of CNS		Proventriculus	
			Crop	
DILP 3	IPCs	IPCs	Corpora cardiaca	Rulifson et al., 2002; Brogiolo et al., 2001; Veenstra et al., 2008
		Muscle cells of midgut	Anterior aorta	
			Proventriculus	
			Crop	
DILP 4	Anterior midgut	–	–	Brogiolo et al., 2001
DILP 5	IPCs	IPCs	Corpora cardiaca	Rulifson et al., 2002; Brogiolo et al., 2001; Söderberg et al., 2011
	Principal cells in renal	Follicle cells of ovary	Anterior aorta	
	Tubules	Principal cells in renal	Proventriculus	
		Tubules	Crop	
DILP 6	Adipose cells	Adipose cells	–	Okamoto et al., 2009; Slaidina et al., 2009
	Salivary glands			
	Heart			
	Glial cells of CNS			
DILP 7	Abdominal neuromeres	Abdominal neuromeres	Brain neuropil	Miguel-Aliaga et al., 2008; Yang et al., 2008
			Hindgut
			Reproductive tract
DILP 8	Imaginal discs	Ovary	–	Garelli et al., 2012; Colombani et al., 2012; FlyAtlas gene expression data base shows ovary expression

IPCs, insulin producing cells.

**Figure 2:**
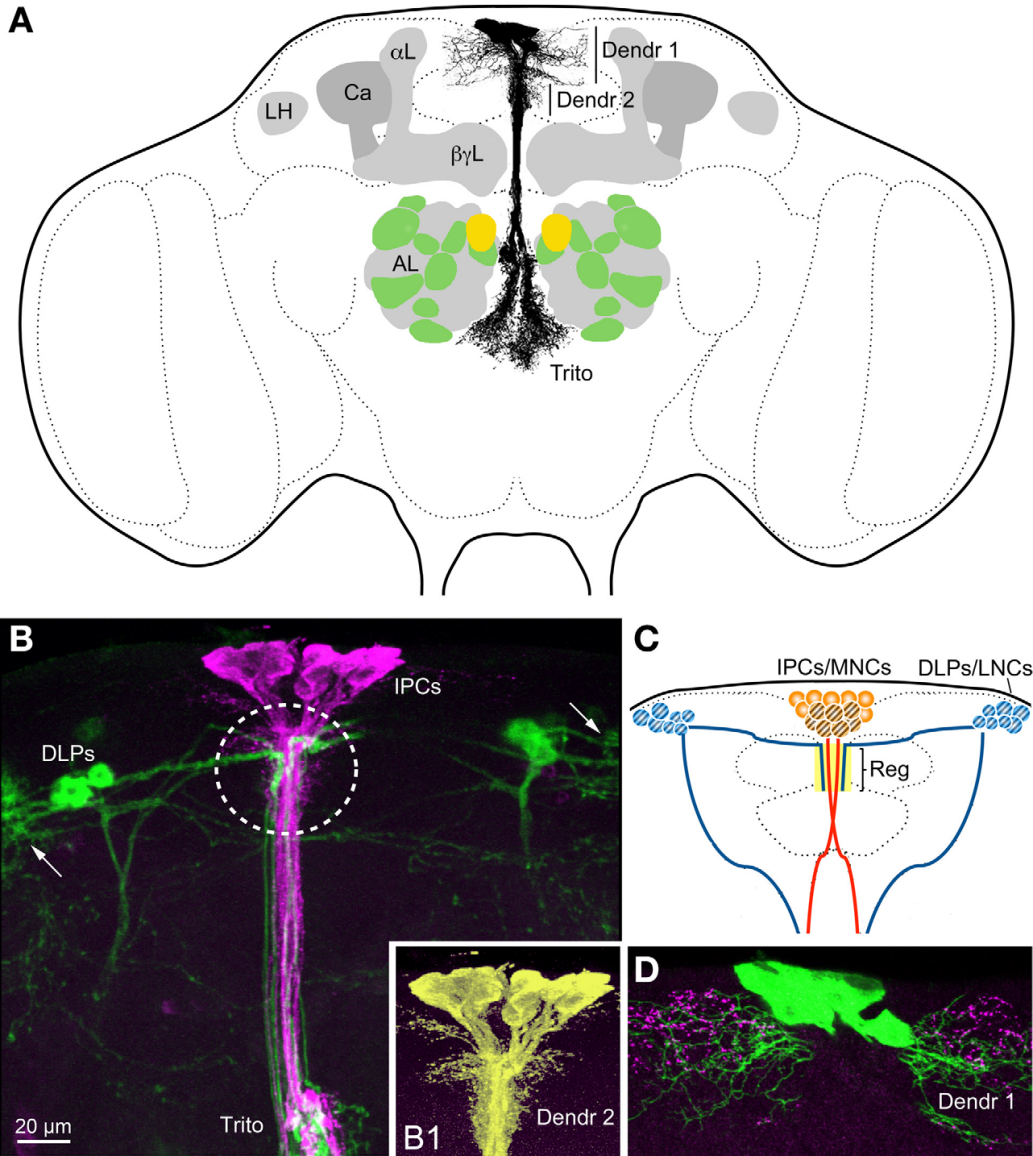
**Insulin-producing cells (IPCs) and other neurons in the *Drosophila* brain. (A)** The IPCs are seen with their cell bodies dorsally, two sets of presumed dendrites (Dendr 1 and 2) in the pars intercerebralis and processes branching in the (tritocerebrum Trito). It is not known whether these branches are dendrites or axon terminations, or both. The axons that exit to the corpora cardiaca and aorta are not displayed (they exit above the tritocerebrum, in a direction toward the reader). The antennal lobes (AL) are depicted with the anterior 10 (green and yellow) of the about 14 glomeruli that contain olfactory sensory neurons (OSNs) expressing short neuropeptide F (sNPF). The yellow glomeruli are DM1 that receive OSNs expressing odorant receptor Or42b and sNPF, known to be essential for food search. These sNPF-expressing OSNs also express the insulin receptor (dInR) and the sNPF receptor. DILPs are known to modulate odor sensitivity of these OSNs (Root et al., 2011). The mushroom bodies with calyx (Ca), α-, β- and γ-lobes (α L, β γ L) and the lateral horn (LH) are also depicted. The mushroom bodies also seem to be targeted by DILPs, at least in larvae (Zhao and Campos, 2012). **(B)** The IPCs (magenta, anti-DILP2) and corazonin-expressing DLP neurons (GFP, green) converge medially in the pars intercerebralis (encircled) and in the tritocerebrum (Trito). The DLPs are known to regulate IPC activity (Kapan et al., 2012). Arrows indicate the likely dendrites of the DLPs. **(B1)** Detail of IPCs (enhanced color) visualizing the short dendrites (Dendr 2) that seem to receive inputs from DLPs. **(C)** Schematic depiction of IPCs, DLPs and their point of convergence in the pars intercerebralis (Reg). The IPCs are located in the MNC cluster and the DLPs among the LNCs. For further details see Figure 3. **(D)** The IPCs (green) may receive inputs from serotonin-producing neuron branches (magenta) both at the long dendrites (Dendr 1) and the short (not shown here). Panel **(B)** is altered from Kapan et al. (2012) and 2D from Luo et al. (2012).

The 14 brain IPCs (Figures 1, 2A) are embedded in a cluster of median neurosecretory cells (MNCs) in the pars intercerebralis (PI). The morphology of the brain IPCs has been described from immunolabeling with several DILP antisera and from use of *Dilp2*-Gal4-driven GFP and it seems that each of the 14 brain IPCs coexpresses DILP2, 3 and 5 (Brogiolo et al., 2001; Cao and Brown, 2001; Ikeya et al., 2002; Rulifson et al., 2002; Broughton et al., 2005; Geminard et al., 2009). In larvae the IPCs additionally produce DILP1 (Rulifson et al., 2002). Recently it was shown that many of the brain IPCs also coexpress drosulfakinin (DSK), a cholecystokinin-like peptide (Söderberg et al., 2012). Available data suggest that these IPCs all share the same morphology. They have cell bodies located in the PI, two sets of branches in the PI, another set in tritocerebrum and axons extending to varicose terminations in the corpora cardiaca, anterior aorta, proventriculus and crop (Figures 1, 2). However, no attempts have been made so far to analyze individual IPCs, which leaves the possibility that some of the 14 neurons have more restricted morphologies. The dendrites, or at least major input sites, of the IPCs have not been identified conclusively in any insect. Thus, it is not known whether the branches in the PI and tritocerebrum (Figure 2) are dendritic or maybe a mix of input sites and peptide release sites. In published accounts antisera to DILPs immunolabel both the IPC branches in the PI and in the tritocerebrum, which suggests that DILPs are stored and maybe released within the brain. In other words it is possible that DILPs are released both into the circulation from neurohemal releases sites and in a paracrine fashion within the brain. A recent study indeed suggests that at least DILP2 is released within the brain neuropil and acts on other neurons of the larval brain (Bader et al., 2013).

There are also a few reports on *Dilp2*-Gal4 expressing neurons in the larval and adult abdominal and thoracic ganglia (Kaplan et al., 2008; Agrawal et al., 2010). However, the expression of DILP2 peptide or *Dilp2* transcript in these cells has not been confirmed, so it is possible that this extra *Dilp2*-Gal4 expression lacks fidelity.

**Figure 3:**
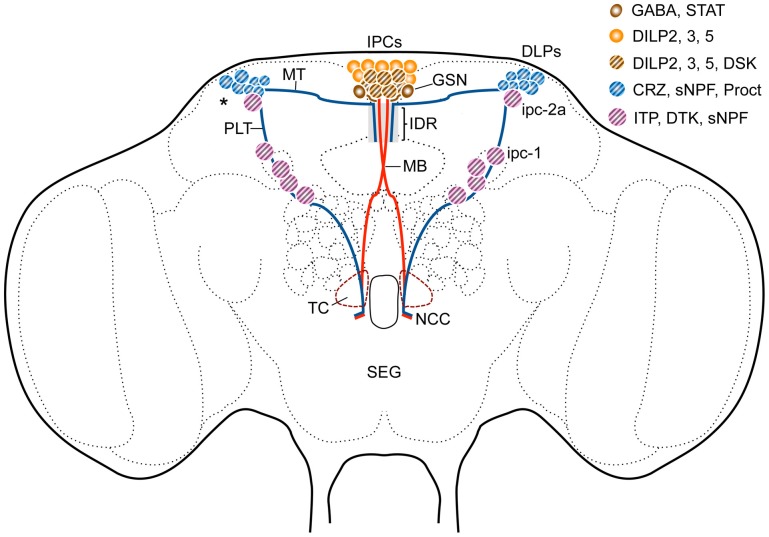
**Sets of peptidergic neurons in the *Drosophila* brain that are associated with the IPCs.** Some of the IPCs co-express drosulfakinins (DSK1 and 2), peptides that induce satiety (Söderberg et al., 2012). The IPCs are regulated by DLP neurons that produce short neuropeptide F (sNPF), corazonin (CRZ) and proctolin (Proct) (Isaac et al., 2004; Kapan et al., 2012) as well as GABAergic neurons (GSN) that in turn receive leptin-like (Upd2) signals from the fat body (Rajan and Perrimon, 2012). The GSNs express a Upd2-activated Jak/Stat receptor (Dome). The interactions between DLPs and GSNs and IPCs probably occur dorso-medially (IDR) in the pars intercerebralis and maybe in the tritocerebrum (TC). Another set of lateral neurosecretory cells (LNCs), designated ipc-1 and ipc-2a, express the peptides sNPF, *Drosophila* tachykinin (DTK) and ion transport peptide (ITP) (Kahsai et al., 2010). These neurons, like the DLPs, are parts of the LNC clusters and have axon terminations in sites overlapping those of the IPCs and the DLPs in the corpora cardiaca and anterior aorta. Further abbreviations: STAT, Jak/stat receptor Dome; MT, medially projecting axon tract; PLT, posterior lateral axon tract; MB, median bundle; NCC, nerves to corpora cardiaca. Asterisk indicates region where dendrites of DLPs arborize.

DILP7 is produced in at least two types of neurons in the ventral nerve cord (Figure 1). There are several sets of DILP7 neurons (dMP2) in abdominal neuromeres A6–9, some of which are efferent with axons that terminate on the hindgut. One pair of DILP7 expressing interneurons (DP) in A1 arborize in the abdominal neuropil and send axons to the brain (Miguel-Aliaga et al., 2008). In the third instar larva the axons of the DP neurons terminate close to the protocerebral branches of the IPCs (Miguel-Aliaga et al., 2008; Nässel et al., 2008) and in the adult brain these axons impinge on the ventral portion of the tritocerebral processes of the IPCs (Cognigni et al., 2011). The DPs, but none of the other DILP7-expressing neurons, coexpress short neuropeptide F (sNPF), and weak *Cha*-Gal4 expression, that indicates presence of choline acetyltransferase and thus suggests a cholinergic phenotype (Nässel et al., 2008). It is therefore possible that the larval DPs modulate the IPCs by means of DILP7 and sNPF (and possibly acetylcholine). The DILP7 producing neurons are likely to release peptide in a paracrine fashion within the CNS and at the hindgut structures (Miguel-Aliaga et al., 2008; Cognigni et al., 2011). A role of DILP7 has also been detected in reproductive behavior in egg laying, correlated with DILP7 containing axons innervating the female reproductive tract (Yang et al., 2008).

It can be noted that the IPCs are a subpopulation of the MNCs and that other MNCs with similar morphology produce distinct neurohormones such as the peptides myosuppressin, and diuretic hormones 31 and 44 (Park et al., 2008). In addition there are bilateral clusters of peptide-producing lateral neurosecretory cells (LNCs; Figure 3) that also send axons to the corpora cardiaca/allata, aorta and anterior gut structures (often referred to as the retrocerebral complex). The LNCs in some cases have collateral processes that superimpose the proto- and tritocerebral processes of the median neurosecretory cells (Figures 2B,C, 3) or converge with them in the retrocerebral complex (see Homberg et al., 1991; Shiga et al., 2000; Siegmund and Korge, 2001; Hamanaka et al., 2007; Kapan et al., 2012). Several of the LNCs produce peptides that are released as circulating hormones, others may produce peptides that act more locally as regulators of release from MNCs (detailed in the next section). Among the peptide hormones produced by LNCs in *Drosophila* are prothoracicotropic hormone (PTTH; in larvae only), corazonin, and ion transport peptide (ITP) (McBrayer et al., 2007; Dircksen et al., 2008; Lee et al., 2008a; Kapan et al., 2012). Thus, the MNCs and LNCs constitute groups of neurons that may play roles reminiscent of hypothalamic neurons of vertebrates (Scharrer, 1972, 1987; Hartenstein, 2006).

## Brief summary of functional roles of IPCs and DILPs

There is an extensive literature on the functional roles of DILPs and more specifically DILPs released from the brain IPCs (reviewed by e. g., Géminard et al., 2006; Giannakou and Partridge, 2007; Grönke et al., 2010; Teleman, 2010; Antonova et al., 2012; Itskov and Ribeiro, 2013). Hence, only a brief summary is provided here to put the subsequent discussion into context. Developmental aspects of DILP signaling (including growth) are not considered here.

Genetic ablation of IPCs, or other manipulations that diminish DILP signaling from these cells affect carbohydrate and lipid metabolism. Thus, fasting glucose levels in the hemolymph increase after IPC ablation, as seen also in diabetic mammals (Rulifson et al., 2002; Broughton et al., 2005). Assays of stored carbohydrates in whole body extracts revealed an increase in both trehalose and glycogen (Broughton et al., 2005). Also triacylglycerol stores increase in flies with decreased IPC activity (Broughton et al., 2005; Slack et al., 2010). Interestingly depletion of DILP2, but not DILP3 and 5 in IPCs, affects stored trehalose, but not the other circulating or stored compounds, suggesting compensation by the other two DILPs (Broughton et al., 2008). Indeed, DILP2 knockdown leads to increased levels of DILP3 and 5.

One of the early findings on insulin signaling in *Drosophila* was that diminished insulin-receptor activity increases lifespan (Clancy et al., 2001; Tatar et al., 2001). It is sufficient to ablate the IPCs to extend both median and maximal lifespan of flies (Broughton et al., 2005). Mated females extended their median lifespans by 33.5% and males by 10.5%. The mortality started later in life of aging IPC-deleted flies, but thereafter at the same rate as in control flies (Broughton et al., 2005). There seems to be a link between dietary restriction, extended lifespan and functional IPCs (Broughton et al., 2010). In control flies a diluted protein (yeast) content in the food extends lifespan by 12–20%. However, IPC ablation renders flies less responsive to dietary restriction in terms of longevity (Broughton et al., 2010).

Responsiveness to stress is likely to, at least partly, be related to lifespan. Oxidative stress certainly appears to be a factor that affects longevity (Broughton et al., 2005; Kenyon, 2005). Diminished signaling from IPCs increases resistance to oxidative stress (Broughton et al., 2005) and it is known that Jun-N-terminal Kinase (JNK) signaling in IPCs is required for adaptive responses to stress (Karpac et al., 2009). A panel of single and combinatory *Dilp* mutants was tested for stress resistance. It was found that only *Dilp2,3,5* and *Dilp1-4* mutant flies display resistance to paraquat-induced oxidative stress, suggesting a requirement of DILP2 and 3 for the stress response (Grönke et al., 2010). Resistance to starvation (and dry starvation) is also dependent on DILP signaling and the IPCs, and flies display increased resistance after inactivated signaling (Broughton et al., 2005); starved *Dilp1-4* mutants display increased survival by 18% (Grönke et al., 2010). On the other hand, the resistance to temperature stress did not increase after diminished insulin signaling (or IPC activity): recovery from cold and heat knockdown was tested (Broughton et al., 2005).

Fecundity is dependent on intact DILP signaling from the IPCs and probably the fat body (Broughton et al., 2005; Grönke et al., 2010). These authors found that ablation of IPCs or genetic diminishment of DILPs 2, 3, 5, and 6 reduce life-time fecundity. Generally, diminished systemic insulin signaling increases life span on the cost of fecundity.

Foraging and feeding are in several ways dependent on DILP signaling. At the sensory level DILPs play a role in regulating strength of odor signals via action on dInR expressed in olfactory sensory neurons (OSNs) of the antennae. It was shown that the receptor of the peptide sNPF (sNPFR) is down-regulated in OSNs in fed flies where circulating insulin levels are increased (Root et al., 2011). The diminished sNPFR expression presynaptically in OSNs decreases synaptic transmission to second order olfactory neurons and hence sensitivity to food odors. This renders the flies less attracted to food sources and food search diminishes. Antennal lobe structures (glomeruli with sNPF/sNPFR expressing OSNs) are shown in Figure 2A. The next level of DILP action is in brain circuits where *Drosophila* neuropeptide F (NPF) and its receptor (NPFR) play important roles in feeding (Wu et al., 2003, 2005a,b). These authors showed that NPF signaling is critical for choice of food in relation to hunger. Also the NPFR is negatively regulated by DILP signaling and interference with the dInR in NPFR-expressing neurons produced behavioral effects on feeding (Wu et al., 2005a,b). Down-regulation of DILP signaling to NPFR neurons leads to a phenotype where fed larvae feed on non-palatable food that is normally rejected, and upregulated DILP signaling induced food aversion in starved larvae. Therefore, it seems that DILPs released at feeding act as a satiety signal via the NPF system (Wu et al., 2005a,b). It has also been shown that silencing or ablating IPCs, and thus decreasing DILP signaling, leads to increased feeding, especially under poor nutritional conditions (Cognigni et al., 2011; Söderberg et al., 2012). Furthermore, sNPF is known to regulate feeding; in part this may be by activating the IPCs and insulin signaling (Lee et al., 2004, 2008b; Hong et al., 2012). This will be further discussed in the next section.

A recent study implicated a role of insulin signaling in feeding by means of action in mushroom body circuits in the brain of *Drosophila* larvae (Zhao and Campos, 2012). Mushroom body structures are shown in Figure 2A. These authors found that knockdown of insulin signaling to intrinsic mushroom body neurons (Kenyon cells) by expression of dominant negative forms of dInR and PI3K (phosphoinositide 3 kinase) reduced food intake in larvae and diminished growth. This diminished signaling did not delay larval development, but affected cell proliferation. However, the mushroom body development, as monitored by size and shape of their lobes, was not affected by the manipulations of PI3k and the downstream FOXO (Zhao and Campos, 2012). If the development of the mushroom body neurons was not affected, then it might be that synaptic activity in this brain center was altered. Since the intrinsic Kenyon cells employ sNPF as a functional neuromodulator (Johard et al., 2008; Knapek et al., 2013) it might be that the sNPF signaling is diminished by insulin action, similar to the antennal OSNs (Root et al., 2011).

There are several indications that insulin signaling plays a role in induction and maintenance of diapause in insects, including species of *Drosophila* (Tatar and Yin, 2001; Hahn and Denlinger, 2011; Antonova et al., 2012; Sim and Denlinger, 2013). When kept at about 11°C and short day conditions *Drosophila melanogaster* females display adult reproductive diapause. This diapause is an overwintering strategy for many insects, characterized by arrested development and reallocation of metabolism and physiology from reproduction to somatic maintenance (Tatar and Yin, 2001; MacRae, 2010; Hahn and Denlinger, 2011). Disruption of various components of the insulin-signaling pathway in *D*. *melanogaster* shuts down reproduction and increases energy stores, inducing a physiologic state similar to the natural adult diapause in other *Drosophila* species (Tatar and Yin, 2001; Salminen et al., 2012). It has also been shown that naturally occurring polymorphisms in genes encoding several insulin signaling components affect diapause induction (Williams et al., 2006; Fabian et al., 2012). The mechanisms of DILP and juvenile hormone signaling in *D. melanogaster* reproductive diapause are yet to be elucidated.

Finally, the IPCs may play a role in sleep regulation (Crocker et al., 2010). These authors found that activation of the IPCs by expressing a constitutively active depolarizing sodium channel reduced night-time sleep and conversely a hyperpolarizing potassium channel decreased sleep. They also showed that an octopamine receptor (OAMB) expressed by the IPCs mediates this effect on sleep, such that octopamine has a wake-promoting effect (Crocker et al., 2010). A null mutation in the OAMB receptor results in increased sleep, and a specific rescue with wild type OAMB only in the IPCs restored normal sleep levels. This study, however, did not test whether DILPs play a role in the regulation of sleep, but a later paper actually shows that DILPs do not mediate the octopamine-effects on sleep/wake (Hong et al., 2012). It can be noted that the IPCs, and maybe even insulin signaling, seem to be of importance for general locomotor activity in flies (Belgacem and Martin, 2002, 2006; Jones et al., 2009).

The above examples of various roles of IPCs and actions of DILPs beg the question: how are the IPCs and DILP release regulated? Can we identify circulating signals that act on the IPCs, and are there neuronal pathways directly devoted to regulation of these cells?

## What controls release of IPCs from CNS neurons?

A key trigger of DILP release from IPCs in feeding stages of *Drosophila* appears to be intake of nutrition. The post-feeding increase in circulating glucose and amino acid levels is sensed by the fat body and as a consequence signals are released into the circulation and reach the brain and the IPCs. This was first studied in larvae where nutrient sensing occurs in the fat body and a fat body-secreted factor activates DILP release (Geminard et al., 2009). The fat body nutritional sensor is the amino acid transporter slimfast, which activates the TOR (target of rapamycin) pathway (Colombani et al., 2003). In the adult fly a similar mechanism has been proposed, and the fat body-derived factor identified as a leptin-like molecule, unpaired 2 (Upd2), that indirectly activates the IPCs by lifting a tonic inhibition from specific GABAergic neurons (Rajan and Perrimon, 2012). This will be dealt with in more detail below. The IPCs do not seem to display clear cell-autonomous nutrient sensitivity, in contrast to the larval secretory cells producing the glucagon-like adipokinetic hormone (AKH) (Kim and Rulifson, 2004). Some evidence, however, exists that the adult IPCs have autonomous glucose sensing by means of glucose uptake, glycolysis and subsequent ATP inactivation of ATP-sensitive potassium (K^ATP^) channels similar to mammalian pancreatic beta cells (Kreneisz et al., 2010). However, no coupling to regulation of DILP release was made in that study. Another membrane-associated channel has been implicated in adult IPCs in regulation of DILP signaling. This is the calcium-activated potassium channel Slowpoke (SLO) that is known to be regulated by a binding partner, the SLO-binding protein (SLOB) (Sheldon et al., 2011). Both SLO and SLOB are expressed by the IPCs, and when their expression is diminished in these cells, *Dilp3* transcript level decreases, energy is stored, and DILP signaling is changed. It was proposed that SLO and SLOB modulate the action potential duration in the IPCs and thus have a possible role in release of DILPs (Sheldon et al., 2011).

A novel nutrient sensor was revealed recently: the fructose sensitive gustatory receptor Gr43a (Miyamoto et al., 2012). In addition to its expression in sensory cells of the proboscis, Gr43a was found in four pairs of neurons in the region of LNCs of the brain. The Gr43a expressing brain neurons can sense circulating fructose and were shown to regulate food intake in a satiety-dependent manner (Miyamoto et al., 2012). Fructose levels increase drastically and transiently in the circulation after a carbohydrate meal, although glucose and trehalose are the predominant hemolymph carbohydrates (Miyamoto et al., 2012). These authors propose that dietary sucrose can be converted to fructose and glucose in the fly and also that fructose is a normal component in *Drosophila's* fruit diet. Possibly the Gr43a-expressing neurons interact with neurons in the LNC cluster that in turn affect feeding circuits or maybe insulin signaling via IPCs.

Recently a receptor resembling the mammalian adiponectin receptor was identified in *Drosophila* IPCs (Kwak et al., 2013). It was proposed that this receptor regulates IPC activity, DILP2 release and that it may respond to another adipokine signal from the fat body. These authors could, however, not identify a adiponectin-like ligand in *Drosophila*.

**Figure 4:**
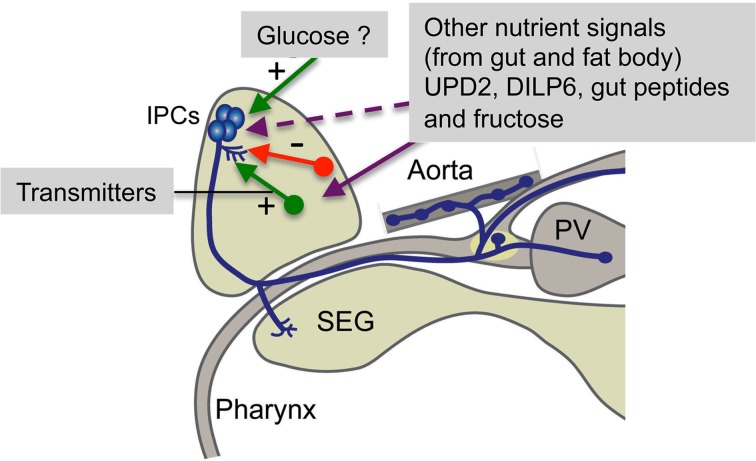
**Summary of factors regulating brain IPCs.** The brain IPCs are regulated by neurotransmitters (including neuropeptides) from neurons in the brain (red and green arrows) in a stimulatory (+) or inhibitory (−) fashion. These neurons are in turn likely to be activated by nutritional signals from the circulation, fat body or intestine. Some of these signals may act directly on the IPCs. UPD2 inactivates GABAergic neurons that then relieves tonic inhibition of IPCs (Rajan and Perrimon, 2012) and circulating fructose acts on brain neurons expressing the fructose receptor Gr43a (Miyamoto et al., 2012). Gut peptides include allatostatin A, DH31 and tachykinins (DTKs), known to have receptors on neurons regulating IPCs or directly on IPCs (Johnson et al., 2005; Veenstra, 2009; Birse et al., 2011). Circulating glucose has been proposed to be sensed by IPCs via uptake, entering glycolysis and the resulting ATP blocking ATP-sensitive K^+^ channels on IPCs leading to membrane depolarization and subsequent opening of voltage sensitive Ca^2+^ channels (Kreneisz et al., 2010). PV, proventriculus; SEG, subesophageal ganglion. Not shown here is the expression of an adiponectin-like receptor in IPCs (Kwak et al., 2013). This receptor may be targeted by a hitherto unidentified adipokine signal from the fat body.

In mammals release of insulin is modulated by several hormones and neurotransmitters, as well as feed-back from circulating insulin (Drucker et al., 1987; Aspinwall et al., 1999; Adeghate et al., 2001; Dong et al., 2006; Sonoda et al., 2008). Similarly, the *Drosophila* IPCs appear to be regulated by neurons producing neuropeptides, monoamines and GABA and receive DILP feedback. Roles of sNPF, corazonin *Drosophila* tachykinin, GABA, serotonin and octopamine in IPC regulation are discussed in the following. A summarizing diagram of pathways that regulate IPCs is shown in Figure 4.

A role of sNPF has been proposed in regulation of feeding, growth and insulin production (Lee et al., 2004, 2008b; Hong et al., 2012). Those studies did not clarify which sNPF expressing neurons are responsible for the regulation of IPCs. In the larva there are two candidate systems of sNPF neurons: (1) the two DP neurons of the first abdominal neuromere that express DILP7 and sNPF (Miguel-Aliaga et al., 2008; Nässel et al., 2008), and (2) a set of lateral brain neurons (DLPs; among the LNCs; see Figures 2B,C, 3) that send axonal processes to the presumed dendrite region of the IPCs and to the corpora cardiaca region of the ring gland where the IPC axons terminate (Nässel et al., 2008). In the adult fly the sNPF expressing DLPs coexpress the neuropeptide corazonin and their axons project to the IPC branches in pars intercerebralis, tritocerebrum and corpora cardiaca (Kapan et al., 2012). It was shown by targeted RNA interference that both sNPF and corazonin from the DLPs stimulate the IPCs and thereby affect carbohydrate and lipid levels (Kapan et al., 2012). After sNPF knockdown in DLPs the transcript levels of *Dilp2* and *5* decrease (*Dilp3* not affected), whereas corazonin-RNAi in the same cells does not affect *Dilp* transcription. Thus, the two peptides co-released from DLPs act in different ways on the IPCs, and corazonin additionally appears to be released into the hemolymph to act on the fat body (Kapan et al., 2012). The DLPs have been shown to express receptors for allatostatin A and diuretic hormones 31 and 44 (Johnson et al., 2005; Veenstra, 2009). The former two peptides are produced by endocrine cells of the midgut (Veenstra et al., 2008) and are possibly released into the circulation during feeding to target the DLPs. If this is the case, then the midgut endocrines may act as nutrient sensors or monitor gut distension during feeding (see Miyamoto et al., 2013). It can also be noted here that the fructose sensing Gr43a-expressing neurons in the area of LNCs might be in a position to act directly on the DLPs (Miyamoto et al., 2012).

The next neurotransmitter to be proposed in the regulation of IPCs was serotonin (5-hydroxy tryptamine; 5-HT). Serotonergic neurons were found to express *Drosophila* NS3, a nucleostemin family GTPase (Kaplan et al., 2008). NS3 manipulations affect growth in *Drosophila* via insulin signaling (Kaplan et al., 2008). When an *ns3* mutant was rescued by expressing *ns3* in serotonergic neurons the growth defects were reversed. These authors suggested that NS3 acts in serotonergic neurons in regulation of insulin signaling and thereby control of organismal growth (Kaplan et al., 2008). The direct connection between serotonergic neurons and IPCs was, however, not revealed in the study. We therefore screened for serotonin receptors on IPCs and found that out of the four known receptors in *Drosophila* only 5-HT_1A_ is expressed in these cells (Luo et al., 2012).

Targeted knock-down of the 5-HT_1A_ receptor in IPCs produced phenotypes that suggest an effect on insulin signaling (Luo et al., 2012). Diminished receptor levels decreased survival of flies at starvation and altered lipid levels, but the responses to temperature stress were the opposite of what was expected for a presumed action of an inhibitory 5-HT_1A_ receptor. The 5-HT_1A_ knockdown flies displayed decreased survival at 39°C and required longer to recover from cold coma induced by exposure to 0°C (Luo et al., 2012). This suggested that the receptor normally has a stimulatory effect on insulin signaling, since an earlier study had shown that decreased insulin release resulting from IPC ablation led to decreased tolerance to heat and cold treatment (Broughton et al., 2005). We speculate that the 5-HT_1A_ receptor is indeed inhibitory and inhibits adenylate cyclase (AC) and protein kinase A (PKA) and subsequently inactivates cAMP response element binding protein (CREB) (see Nichols and Nichols, 2008). Activated CREB is known to inhibit insulin signaling (Walkiewicz and Stern, 2009), and therefore inhibition of AC, PKA, and CREB would stimulate insulin signaling. The adverse effect of 5-HT_1A_ knockdown on survival at starvation and on lipid levels could be caused by an increase in locomotor activity as a response to starvation (Lee and Park, 2004), where more energy is consumed and thus overrides the insulin-mediated effect on starvation resistance.

The inhibitory neurotransmitter GABA acts via ionotropic or metabotropic receptors, but only the metabotropic GABA_B_ receptor (GBR) was detected on the *Drosophila* IPCs (Enell et al., 2010). Targeted knockdown of the GBR in IPCs resulted in phenotypes indicating that its role is to inhibit production and/or release of DILPs. Flies with diminished GBR in IPCs displayed shorter lifespan than controls, decreased starvation resistance and altered carbohydrate and lipid metabolism (Enell et al., 2010). The ionotropic GABA_A_ receptor subunit RDL was not found on the IPCs, although it is otherwise very widespread. This study indicated that GABA inhibits activity in the IPCs via its metabotropic receptor, but it was not shown what triggers GABA signaling to the IPCs. The regulation of GABAergic neurons acting on the IPCs was, however, clarified in a later study. It was found that the leptin-like Upd2 (Unpaired 2; related to type 1 cytokines) is released from the fat body after feeding and acts on its receptor Dome that is expressed on a few GABAergic neurons adjacent to the IPCs (Rajan and Perrimon, 2012) (Figure 3). Upd2 activates JAK/STAT signaling in the Dome-expressing GABAergic neurons and thereby lifts the tonic inhibition of the IPCs and allows DILP release. Thus, a nutrient-triggered signal from the fat body acts indirectly via GABAergic neurons to induce systemic DILP signaling. This model requires that Upd2 can pass through the blood-brain barrier to be able to act on GABAergic neurons in the brain.

Another modulatory control of IPCs is by means of peptide products of the *Drosophila* tachykinin (DTK) precursor gene (*Dtk*). Six DTK peptides have been identified, five of which are expressed in the CNS (Siviter et al., 2000; Winther et al., 2003). We found that one of the two known DTK receptors, DTKR, is expressed in IPCs and that knockdown of this receptor affects the IPCs (Birse et al., 2011). Diminishment of DTKR expression on IPCs results in decreased starvation resistance, and a more rapid decrease of whole body trehalose, but has no effect on lipid levels. These results suggest that also DTKR inhibits insulin signaling from the IPCs. An important finding in this context was that DTKR knockdown in IPCs affects *Dilp* transcript levels in brains of fed and starved flies. It could be shown that only *Dilp2* and *Dilp3*, but not *Dilp5* transcripts were affected by receptor knockdown (Birse et al., 2011). After DTKR-RNAi the *Dilp2* and *3* transcripts both increased in fed flies, whereas after 24 h starvation the *Dilp3* transcript decreased and *Dilp2* increased. This suggests that the DTKR activation induces transcriptional effects that are differential for the three *Dilp* genes. Similar individual regulation of *Dilp* transcription in the brain IPCs has been shown in multiple other experiments: for instance after nutritional restriction, manipulations of JNK signaling or SLO/SLOB expression in IPCs, NS3 in serotonergic neurons and sNPF in DLP neurons (Broughton et al., 2008, 2010; Kaplan et al., 2008; Karpac et al., 2009; Sheldon et al., 2011; Kapan et al., 2012). We will return to the possible role of differential transcriptional control of *Dilps* in the concluding section.

A further neurotransmitter/neuromodulator that has been suggested to modulate IPC activity is octopamine (Crocker et al., 2010). It was shown that the IPCs express one of the octopamine receptors, OAMB, and knockdown of this receptor altered sleep-wake patterns in the flies. Stimulation of the IPCs with octopamine increased cAMP in these neurons and the wake-promoting effect of octopamine appears to be dependent on PKA activation in the IPCs (Crocker et al., 2010). These authors, however, did not provide any evidence that octopaminergic activation of the IPCs affects systemic insulin signaling.

There is some evidence that various DILPs may provide feedback onto the brain IPCs. Experiments show that overexpression of *Dilp6* in the fat body of adult flies leads to extended lifespan, but also to a repression of brain levels of *Dilp2* and *5* transcript and a decrease in circulating DILP2 levels (Bai et al., 2012). These authors suggest that DILP6 release from the fat body may act locally on dInR in the fat body (in autocrine fashion), but also repress IIS in other tissues by inhibiting DILP2 release from the IPCs. This would suggest an inhibitory action of DILP6 on IPCs directly or indirectly via other neurons. The latter is possible since increased expression of DILP6 in fat body suppresses sNPF expression in the brain (Bai et al., 2012), and sNPF is known to regulate the IPCs and *Dilp* transcription (Lee et al., 2008b; Kapan et al., 2012). Another set of experiments suggests that DILPs released from the IPCs may feed back to the IPCs. Knockdown of *Dilp2* leads to an increase in *Dilp3* and *5* mRNA, and via other manipulations it was shown that at least *Dilp3* transcription is regulated via autocrine DILP action (Broughton et al., 2008). This autocrine or paracrine regulation of *Dilp3* (via the dInR and FOXO) may involve DILP2, 3 and 5 (Broughton et al., 2008; Grönke et al., 2010). As mentioned earlier there might also be a communication between IPCs and two neurons in the abdominal ganglion expressing DILP7 (Miguel-Aliaga et al., 2008; Cognigni et al., 2011), but the functional connections have not yet been demonstrated.

Finally, it has been suggested that there may be direct functional connections between IPCs and cells that produce AKH in the corpora cardiaca (Rulifson et al., 2002; Buch et al., 2008). Ablation of AKH-producing cells leads to increased *Dilp3* mRNA, but unchanged levels of *Dilp2* and *5*, and ablation of IPCs resulted in increased Akh transcript (Buch et al., 2008).

At present the only modulatory neuronal pathway that has been placed in a functional context with respect to systemic insulin signaling is the GABAergic one (Rajan and Perrimon, 2011). Here it was shown that food intake leads to the release of a fat body-derived leptin-like factor that triggers inhibition of specific GABAergic neurons that in turn lift a tonic inhibition of the IPCs. This disinhibition presumably facilitates DILP release. It is important for the near future to investigate how the other peptidergic and aminergic neuronal systems are targeted by systemic signals that reflect levels of nutrients, stress and other factors that trigger or inhibit insulin signaling. There are some additional proteins that recently have been implicated in regulation of DILP release from IPCs in *Drosophila* that also need to be linked to upstream systemic signals: an adiponectin receptor and the protein interacting with C kinase 1 (PICK 1), both of which are expressed in IPCs (Holst et al., 2013; Kwak et al., 2013).

## Control of release of DILPs from glial cells and other sources

Above we have discussed DILP producing neurons in the brain and how they may be regulated. What about DILP production/release in other cell types? It has been reported that during larval development DILP2 and 6 are expressed in glial cells of the ventral nerve cord (Chell and Brand, 2010). Some of these glial cells are located at the surface of the ventral nerve cord, but underneath the basement membrane (i.e., inside the blood brain barrier). The DILP producing glial cells are in contact with neuroblasts (neuronal stem cells) and it was shown that DILPs via the dInR and PI3K reactivate these dormant neuroblasts in a nutrient dependent manner (Chell and Brand, 2010; Sousa-Nunes et al., 2011). *Dilp6* transcription increases in the glia of larvae 24 h post-hatching and is dependent on amino acids in the food. Thus, for increase in DILP expression, and its paracrine release, a nutrient-dependent signal from the fat body is required to act on the glial cells (Chell and Brand, 2010; Sousa-Nunes et al., 2011). This signal obviously has to pass through the blood-brain barrier, similar to the leptin-like Upd2 described earlier (Rajan and Perrimon, 2012) and mechanisms for such a passage are not known in *Drosophila*.

Another case of nutrient-dependent activation of stem cells by paracrine DILP release was demonstrated in the *Drosophila* intestine (O'Brien et al., 2011). In the adult fly midgut stem cells proliferate and thus ensure growth of the intestine under good nutritional conditions. It was shown that DILP3 is expressed by muscle fibers in a region of the midgut rich in stem cells (Veenstra et al., 2008; O'Brien et al., 2011). The expression of *Dilp3* transcript increases in fed flies during adult gut growth, and in a temporal fashion matching the dynamics of stem cell proliferation. Experiments showed that DILP3 in the stem cell niche is necessary for cell proliferation (O'Brien et al., 2011). These authors propose that DILP3 production and release may depend on local nutrient sensing in the intestine, but that circulating DILPs may provide additional activation of gut stem cells.

Finally, it was shown that principal cells of the *Drosophila* Malpighian tubules express DILP5 both in larvae and adults (Söderberg et al., 2011). Malpighian tubules regulate water and ion homeostasis, but may also play roles in immune responses and oxidative stress (Dow, 2009). The DILP5 levels in the principal cells are dependent on desiccation stress, but also on signaling by tachykinins (DTKs) via their receptor DTKR (Söderberg et al., 2011). Thus, DTKR is expressed in principal cells and so is the dInR. Targeted knockdown of DTKR, DILP5 or the dInR in principal cells or mutation of *Dilp5* resulted in increased survival at desiccation, starvation and oxidative stress, whereas over-expression of these components produced the opposite phenotype (Söderberg et al., 2011). Therefore, various stressors seem to induce hormonal release of DTKs from the intestine that act on the renal tubules to regulate local DILP5 signaling and thus functions of Malpighian tubules related to overcoming oxidative stress.

## Conclusions and outlook

The IPCs of the brain have a central role at the interface between nutritional status of the fly and control of homeostasis, physiology, and behavior. Systemic insulin signaling therefore regulates a wide array of functions in the daily life of an organism, including responses to various types of stressors. Importantly IPCs, and other sources of DILPs, also regulate mechanisms in growth and aging, and thereby life span. DILP signaling can be both systemic and paracrine. Systemic DILP signaling is primarily by means of the IPCs that release DILP2, 3 and 5, or the fat body releasing DILP6 (Brogiolo et al., 2001; Rulifson et al., 2002; Okamoto et al., 2009; Slaidina et al., 2009; Bai et al., 2012). Paracrine signaling has been suggested within the brain for DILP2 from IPCs (Bader et al., 2013), for DILP2 and 6 from glial cells to neuroblasts (Chell and Brand, 2010; Sousa-Nunes et al., 2011), for DILP3 from midgut muscle fibers to gut stem cells (O'Brien et al., 2011) and for DILP5 acting locally in Malpighian tubules (Söderberg et al., 2011).

The IPCs also express the cholecystokinin-like peptides DSK 1 and 2 that seem to induce satiety in flies (Söderberg et al., 2012). Most likely the DILPs and DSKs act in a synchronized fashion on different targets after onset of feeding. However, the IPCs have been implicated in some activities that might not necessarily involve release of DILPs into the circulation. For instance the sleep-wake regulation by octopaminergic inputs to the IPCs (Crocker et al., 2010) could be by means of either paracrine release of DILPs into the brain or by central release of some other colocalized substance (DSK or other neurotransmitters). Another likely paracrine action of DILPs is in regulation of NPF expressing neurons in feeding circuits (Wu et al., 2005a,b). Further indication of paracrine CNS action is the recent finding that DILP2 activates a small set of identified neurons in the brain, as monitored by PKB phosphorylation (Bader et al., 2013). These brain neurons responding to DILP2 include the hugin-expressing neurons known to regulate feeding. The hugin neurons also take up DILP2, probably via dInR internalization, and express an insulin binding protein (Imp-L2). These examples of possible paracrine DILP signaling in the brain suggest roles in modulation of behavior that involve various central neuronal circuits and clearly deserve further study. Can we expect that the dInR is expressed in neurons of dedicated brain circuits, as seems to be the case in mammals (Laron, 2009; Fernandez and Torres-Aleman, 2012)?

A somewhat puzzling feature of IPCs is that they co-express three different *Dilp* genes that are subject to individual transcriptional regulation. Different stimuli affect the transcription of these three genes in a multitude of combinations. However, most likely a depolarization of the IPCs induces release of all the peptides in whatever stoichiometric ratio they are stored. Since the DILPs 2, 3 and 5 have partly redundant functions, and only one form of dInR has been clearly identified (Brogiolo et al., 2001; Broughton et al., 2008; Grönke et al., 2010) one may wonder why the ratio between DILPs is variable. Do the individual DILPs have distinct functions? To test this it is important to investigate whether there are additional dInR subtypes, as in mammals, or if the different DILPs at least have different affinities for, or activities, on the single known receptor.

Finally, it is important to obtain an integrated view of the neuronal systems that regulate the IPCs and the physiological conditions that induce or inhibit release of DILPs. At present we know of several sets of neurons that utilize GABA, monoamines and neuropeptides to regulate activity in the IPCs, but only for the GABAergic neurons we have some clues what triggers the system. Therefore, it is important to further analyze the anatomy of the neuronal circuits that target the IPCs and to search for circulating (and other) signals that use these circuits to mediate aspects of the metabolic and physiological status of the organism.

### Conflict of interest statement

The authors declare that the research was conducted in the absence of any commercial or financial relationships that could be construed as a potential conflict of interest.
